# Address to Graduating Class of Grady Hospital, 1904

**Published:** 1904-10

**Authors:** C. D. Hurt

**Affiliations:** Atlanta, Ga.


### ADDRESS TO GRADUATING CLASS OF GRADY HOSPITAL, 1904.

By C. D. HURT, M.D.,

ATLANTA, GA.

*Mr. President, Ladies and Gentlemen:*

The occasion which brings us together this afternoon is one annually repeated in this institution. This, the Grady Hospital Training School for Nurses, has been sending out annually a class of young ladies equipped for this important work.

The general public does not fully understand the great efforts which have to be made by those young ladies who seek to become trained nurses. Some are ready to imagine that it is but an idle fancy, a light duty and a flippant life. Others may imagine that the accomplishments, when all told and best attained, scarely secure for these nurses the honor of graduation, the acquirement of scientific knowledge, the qualifications for work through which humanity is to be raised from its sufferings, restored from disease to health, habilitated with a sound mind and sound body. Who does not see in the daily walks of life some class of suffering, and any individual of a sympathizing heart and mind naturally desires to see relief for such. Manifestations made by those who are well, in rushing to one injured or sick, falling upon the street, demonstrates the tenderness of the human heart. For six thousand years humanity has been subject to ills and ailments. We need only to look at the advancements in the science of medicine to show what great progress has been made and what relief is to-day offered in lieu of the ignorance, the superstition, I might say folly, of some experiences in the crude ages. All along through these thousand of years; when sickness grew serious in any family or community, there were ever ready sympathizers to offer assistance, comfort and relief. That was but natural, and many did the best that could be done with the knowledge and lights then before them. With the advancement in every science, including that of training for nursing, light and knowledge have been opened up, and to-day we no longer as medical men in making our rounds for the sick, leave them in the hands of untrained, uneducated and unqualified nurses. The doctor to-day is strengthened in his position, supported and emboldened in his work, on account of a knowledge of the fact that when he is not present with the patient, he leaves a faithful and accomplished assistant in that of a trained nurse. Therefore, with all the advancement in civilization, in sciences, and in the knowledge of suffering, the trained nurse has come to be a necessity. Who are trained nurses? Who are qualified to make faithful, efficient helpers in the time of sickness and suffering? The young woman who decides to adopt this profession ought wisely to consider the sacrifices which she will be called upon to make; of her leaving home and friends, surrounded with the comforts and associations which, in a degree at least, are calculated to make her happy; that of the self-denial which she has to endure and the hardships which confront her in placing herself under the discipline of a training school where rules of government are rigid, where duties are hard and irksome, where tasks require the toil of the day, and sometimes of the night; where criticism may be harsh; and for a term of years to bear all of these before she can wear the badge of full qualification.

She must contemplate thoughtfully, carefully, what is in store for any one who embarks in such an important and laudable mission, and study well her own temperament and disposition to meet the issues in this manner of life. She must further calculate that the field of a trained nurse is not strewn with roses, owing to the adverse criticism of suffering humanity desiring to be relieved; of anxious friends imagining that more can be accomplished; of meddlesome lookers-on who seem to know better than any one else what should be done. She must contemplate what is to be accomplished in this great field of life-work. There is a bright side to it; there are people of gratitude; there are people of tenderness; there are people of consideration and sympathy, even in the hour of severest trial when their loved ones are about to pass from the stage of life under the care of the trained nurse.

Disease often disqualifies the patient for unbiased, conscientious judgment in the management by the trained nurse. Such neurasthenics and hysterical patients are to be pitied and borne with, and managed very judiciously by the trained nurse. This is a part of the training inculcated by lectures and in the hospital clinics to each and every nurse.

All nurses do not succeed; neither do all doctors of medicine, nor all doctors of divinity, nor all doctors of law, or of any science, in the full accomplishment of the work before them; but be it said to the credit of the trained nurses that they take this training, heed the counsels given them in lectures, and learn by experience and observation that they receive the rewards of expressions of gratitude of consciousness of duty, and of steady employment when the work is constantly on their minds and hearts. Many of you who hear me to-day have been made fully aware of the great comfort and help of the faithful trained nurse in the home where sickness dwells. The trained nurse has come to stay. I prefer to be optimistic in my ideas of life, and where some of them have failed, become discouraged, and abandoned the work, thousands are to-day, all over our land and country, laboring hard and faithfully, as well as intelligently, to take care of the sick. A liberal education is of help to a trained nurse; a sound mind and body enables her to perform her duties with intelligence and strength; a moral character, with integrity and truthfulness, are absolutely indispensable to qualify her for the position in life.

Then it behooves the public to regard the profession of the trained nurse as a necessity in this day of advancement of science and of higher civilization, to regard the woman who enters this field of science as one of honesty and integrity, and socially fitted to enter the private homes for the purpose of bringing comfort and help in the hour of sickness, in the hour of greatest trial, during the agonies and suffering of some loved one. The trained nurse is entitled to respect and consideration. While some of her duties are menial and hard, she must be able to say of these duties like the doctor or surgeon who lays hold with all his might in handling the sick, with the idea that there is nothing unclean in medicine or surgery. Individual families, therefore, who expect to receive such benefits to their sick, ought and should recognize the spirit of sympathy and gentle nature, the true womanhood of the faithful nurse who enters their sanctum.

Assuming that such is true, from the experience of the past two or three decades, and that the reversion of thought and consideration in the minds of the public is constantly growing towards a fair consideration of the trained nurse, I predict that the time is near at hand when the nurse shall have accorded to her a deserving consideration from the public at large. To you young ladies of the graduating class, it affords me much pleasure to see you to-day receiving the badge of qualification preparatory to leaving the institution of your training, with an earnest hope on the part of your instructors and your Superintendent of Nurses that the field shall ever be pleasant and fair before you.

You will find when you have had sufficient experience, and this I hope has been impressed upon you, that no detail of the work which you have chosen in life will be so insignificant as to allow you to trespass in discharging these duties. As you go forth to-day, qualified for the work, with the dignity of true womanhood and the embodiment of your science, with skill attained by your years of experience, with a cultivated and well-directed sympathy, with an ability for prompt execution of your work, I commend you. No aspiration which has yet crossed your mind is more laudable than that to seek perfection in the duties of the trained nurse.

You may have had in your thoughtful hours imaginations of the powers and influence of a queen upon her throne, and while the opportunities of those in a regal position may be manifoldly multiplied, they ought not to be more potent for good when taken advantage of by more enterprising ones, nor fill the heart of the possessor with more pleasure than that which shall come to you when you shall go into the home of a loving family and administer to suffering humanity. It is not only your duty, but your highest privilege to relieve actual pain and suffering, and a still higher privilege to offer consolation and condolence to a family in distress. With a heart in you throbbing for the accomplishment of good, you will be able to make your own life happier because of duty well performed. In the pursuit of the work which you have selected you will be thrown in the humble cottage, in the aristocratic home and in the royal palace; in all of these remember that the human heart and human life is none the less dear on account of the environments. You should not be unmindful of the frailties of human nature, of the incidents and accidents which befall humanity in life, nor of the baneful environments which often ward off many of the temporal comforts to the unfortunate. What responsibilities there are! What a high calling is yours! what opportunities does it create with you for doing good! what inspiration touches your heart and mind while you are in the active performance of your legitimate duties! Yours is a dignified calling, young ladies. Many times your duties may be servile and embarrassing, but under no conditions should they be degrading and lowering. As you leave your Alma Mater to-day, with a badge of accomplishments, with a heart for your work, with the courage to meet the issues of life, I commend you to the public. You go forth with the endorsement of the officers of this institution, and as we meet now and then in future years, I trust that all your efforts will tend to the advancement of your science and to your own upbuilding and happiness.

